# Hyperthermic intraperitoneal chemotherapy in patients with incomplete cytoreduction for appendiceal pseudomyxoma peritonei: a 10-year treatment experience in China

**DOI:** 10.1186/s13023-023-02995-w

**Published:** 2024-01-04

**Authors:** Bing Wang, Ruiqing Ma, Guanjun Shi, Xiwen Fan, Benqiang Rao, Hongbin Xu

**Affiliations:** 1 Department of Myxoma, Aerospace Center Hospital, Beijing, 100049 China; 2 Department of Gastrointestinal Surgery, Beijing Shijitan Hospital, Capital Medical University, Beijing, 100038 China

**Keywords:** Hyperthermic intraperitoneal chemotherapy, Incomplete cytoreduction, Pseudomyxoma peritonei, Survival, Progression-free survival

## Abstract

**Background:**

To explore the application value of hyperthermic intraperitoneal chemotherapy (HIPEC) in patients with incomplete cytoreduction for appendiceal pseudomyxoma peritonei (PMP).

**Methods:**

We retrospectively analyzed the clinical data of 526 patients with incomplete cytoreduction for appendiceal PMP to discover its prognostic factors, and the therapeutic value of HIPEC.

**Results:**

The 5-year and 10-year overall survival rates of patients after cytoreductive surgery (CRS) treated with HIPEC were significantly higher than those without HIPEC (5y-OS: 58% vs. 48%, 10y-OS: 37% vs. 16%, P = 0.032). The median progression-free survival (PFS) following CRS was 20 months, with a 20% 3-year PFS. The median PFS following CRS + HIPEC was 33 months, with a 60% 3-year PFS (P = 0.000). Univariate analysis indicated that HIPEC, gender, completeness of cytoreduction (CCR) and pathological grade had statistical difference. Multivariate analysis showed that CRS without HIPEC and high pathological grade were independent risk factors for poor prognosis and rapid tumor progression.

**Conclusions:**

HIPEC may prolong the survival in patients with incomplete cytoreduction for low-grade appendiceal PMP. High pathological grade indicates poor survival and rapid tumor progression.

## Introduction

Pseudomyxoma peritonei (PMP) is a rare disease characterized by mucinous ascites, which leading to abdominal distension and bowel obstruction [1]. It usually originates from the appendix [2]. The combination of cytoreductive surgery (CRS) and hyperthermic intraperitoneal chemotherapy (HIPEC) has been regarded as standard treatment for PMP [3]. CRS aims to remove all visible tumors and includes peritonectomy and resection of adjacent organs [4], which has an affirmed effect [5, 6]. However, the efficacy of HIPEC after CRS in the treatment of incomplete cytoreduction for appendiceal PMP is less reported. Therefore, we retrospectively analyzed our single center experience during the last 10 years, in order to verify the role of HIPEC and provide theoretical basis for clinical application.

## Patients and methods

### Patients

Medical records from a database of patients with PMP who attended the Aerospace Center Hospital, Beijing, China between January 2010 and June 2020 were retrospectively reviewed. Inclusion criteria were: (1) diagnosis of appendiceal PMP on histology and histopathologic subtype confirmed by two experienced pathologists; and (2) CRS with or without HIPEC. Exclusion criteria were: (1) complete cytoreduction for appendiceal PMP (completeness of cytoreduction (CCR) = 0/1); (2) PMP derived from other organs or diseases (e.g., ovary, colon, and pancreas); (4) loss to follow-up; (5) incomplete medical records. A total of 526 patients were included in the final analysis.

### CRS and HIPEC treatment

The median abdominal incision was taken from the xiphoid process to the symphysis pubis. The abdominopelvic cavity was fully explored and the peritoneal cancer index (PCI) was evaluated. According to the evaluation criteria described by Professor Sugarbaker [7], who divided the abdomen, pelvic cavity and small intestine into 13 regions. Each area was scored 0–3 points according to the maximum diameter of the tumor. The highest total score was 39 points.

After PCI evaluation, peritonectomy and organ resection procedures were used to remove all visible tumors as much as possible, as described by Sugarbaker [8]. For patients with severe involvement around the stomach or on the surface of the small intestine that cannot be removed, we focused on solving the problems that have the greatest impact on the patient’s quality of life. We should debulk tumors maximally under the condition of ensuring safety. After CRS, we evaluated the CCR in the abdominopelvic cavity. CCR was scored on a scale from 0 to 3, where CCR-0 was no macroscopic residual cancer remained, CCR-1 was residual tumor nodules < 2.5 mm remained, CCR-2 was nodules between 2.5 mm and 2.5 cm remained, and CCR-3 was persistent tumor nodules > 2.5 cm remained [7]. CCR 2–3 was defined as incomplete cytoreduction.

HIPEC was conducted after CRS and before digestive tract reconstruction for 60 min using a closed-abdomen technique with mitomycin (20 mg/m^2^) or cisplatin (75 mg/m^2^), and an extracorporeal device that maintained intraabdominal temperature between 41 and 42 °C [9]. Elderly patients with unstable intraoperative vital signs that make them unable to tolerate prolonged anesthesia and mesenteric contracture are the reasons that prevent patients from doing HIPEC.

### Follow-up

The patients were reexamined every 6 months, including abdominopelvic enhanced CT and tumor markers. The follow-up method was telephone and or reexamine. The follow-up time was from the operation date to June 2020, and the overall survival (OS) was counted. The progression-free time was from the operation date to tumor recurrence. All patients were followed up. Complications were measured by the Clavien-Dindo postoperative complication rating system as the standard [10].

### Statistical analysis

Statistical analyses were conducted using SPSS 20.0 (SPSS, Chicago, Illinois, USA). Continuous data were presented as medians and range. Categorical data were presented as number and percentages. Univariate survival analysis was performed with the Kaplan-Meier method and the log-rank test. Statistically significant variables were included in a multivariate analysis, which used a Cox proportional hazards model to identify independent prognostic factors for survival and progression-free survival (PFS). All live patients were censored. P < 0.05 was considered statistically significant.

## Results

### Clinicopathological characteristics

Demographic and clinical characteristics of the 526 included patients were presented in Table 1. 226 patients were male (43%) and 300 were female (57%). Patient’s median age at hospitalization for PMP originating from appendix was 59 years (range, 27–85 years). The median OS was 47 months (range, 3–288 months) and the median PFS was 27 months (range, 3–126 months). At the last follow-up in June 2020, 226 (43%) patients were still alive, and the 5-, 10-year survival rates were 50% and 24%, respectively.

PCI was < 30 and ≥ 30 in 195 (37%) and 331 (63%) patients, respectively. CCR score was 2 and 3 in 229 (44%) and 297 (56%) patients, respectively. Operation time was < 480 and ≥ 480 in 240 (46%) and 286 (54%) patients, respectively. Blood loss was < 1000 and ≥ 1000 in 89 (17%) and 437 (83%) patients, respectively. Pathological diagnosis showed 367 (70%) patients had low-grade mucinous carcinoma peritonei (LG-MCP) and 106 (20%) patients had high-grade mucinous carcinoma peritonei (HG-MCP) and 53 (10%) patients had high-grade mucinous carcinoma peritonei with signet ring cells (HGMC-S). There were 407 (77%) patients had received HIPEC and 119 (23%) patients had no HIPEC. Among the patients who underwent HIPEC, 174 (33%) patients used Mitomycin (MMC), 220 (42%) patients used Cisplatin (DDP), and 13 (2%) patients used MMC + DDP. 158 (30%) patients received previous systemic chemotherapy (PSC), and 368 (70%) patients did not receive PSC. 289(55%) patients received previous surgery, and 237(45%) patients did not receive previous surgery.

**Table 1:** Patients’ clinical and demographic data (n = 526)

Characteristics	No. of patients
**Gender**	
Male	226 (43%)
Female	300 (57%)
**Age at hospitalization (years)**	
Median (range)	59 (27–85)
< 50	117 (22%)
≥ 50	409 (78%)
**PCI**	
Median (range)	31 (12–39)
< 30	195 (37%)
≥ 30	331 (63%)
**CCR**	
2	229 (44%)
3	297 (56%)
**Operation time (min)**	
Median (range)	490 (99–872)
< 480	240 (46%)
≥ 480	286 (54%)
**Blood loss (ml)**	
Median (range)	1500 (0-11000)
< 1000	89 (17%)
≥ 1000	437 (83%)
**HIPEC**	
Yes	407 (77%)
No	119 (23%)
**HIPEC drug regimen**	
MMC	174 (33%)
DDP	220 (42%)
MMC + DDP	13 (2%)
**PSC**	
Yes	158 (30%)
No	368 (70%)
**Previous surgery**	
Yes	289 (55%)
No	237 (45%)
**Pathological grade**	
LG-MCP	367 (70%)
HG-MCP	106 (20%)
HGMC-S	53 (10%)

*PCI* peritoneal cancer index, *CCR* completeness of cytoreduction, *HIPEC* hyperthermic intraperitoneal chemotherapy, *MMC* mitomycin, *DDP* cisplantin, *PSC* previous systemic chemotherapy, *LG-MCP* low-grade mucinous carcinoma peritonei, *HG-MCP* high-grade mucinous carcinoma peritonei, *HGMC-S* high-grade mucinous carcinoma peritonei with signet ring cells

### HIPEC related overall survival and progression-free survival analysis

The 5-year and 10-year survival rates of patients after CRS treated with HIPEC were significantly higher than those without HIPEC (5y-OS: 58% vs. 48%, 10y-OS: 37% vs. 16%, P = 0.032). The median PFS following CRS was 20 months, with a 20% 3-year PFS. The median PFS following CRS + HIPEC was 33 months, with a 60% 3-year PFS.

### Univariate analysis

**Fig. 1:**
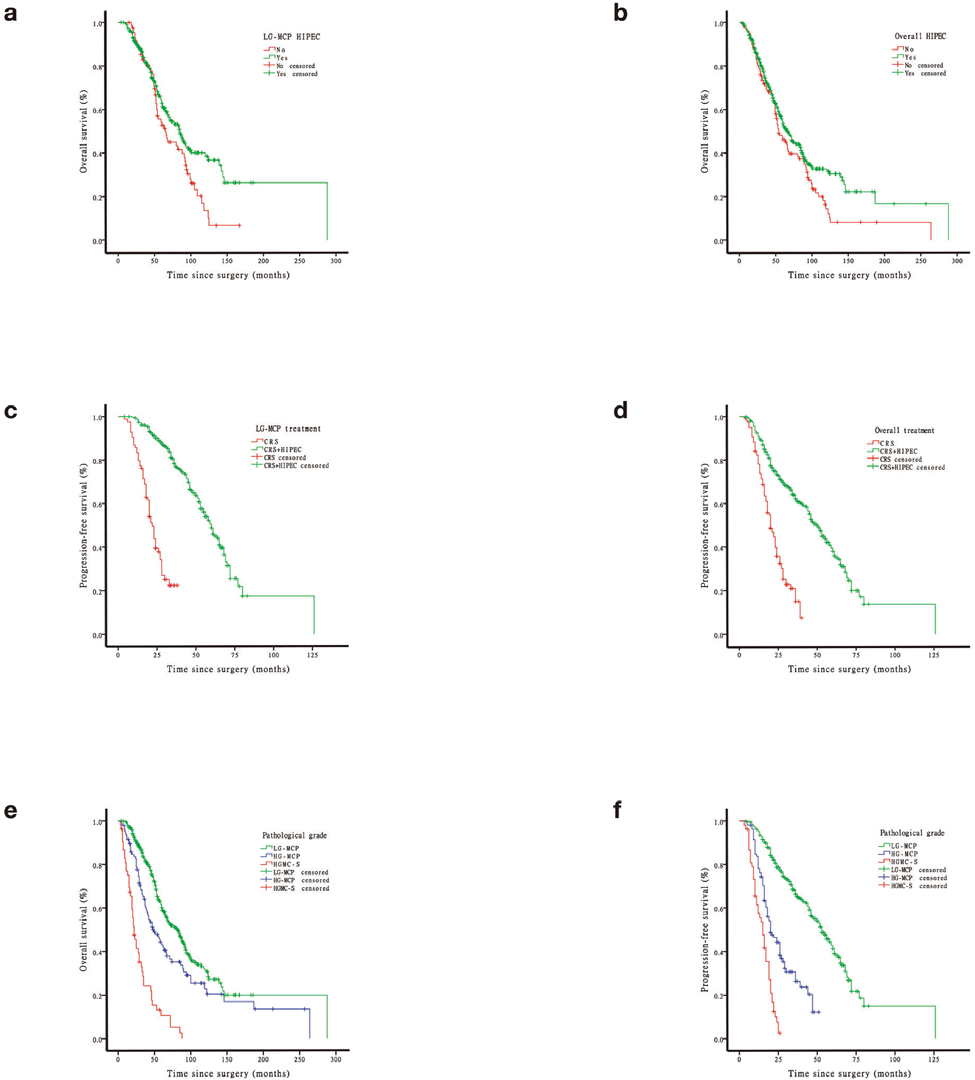
Survival curves and progression-free survival curves in patients with incomplete cytoreduction for appendiceal pseudomyxoma peritonei. (**a**) Survival for LG-MCP patients with or without HIPEC after CRS. (**b**) Survival for all patients with or without HIPEC after CRS. (**c**) Progression-free survival for LG-MCP patients with or without HIPEC after CRS. (**d**) Progression-free survival for all patients with or without HIPEC after CRS. (**e**) Survival for different pathological grade in appendiceal pseudomyxoma peritonei. (**f**) Progression-free survival for different pathological grade in appendiceal pseudomyxoma peritonei

Prognostic factors for OS on univariate analysis were presented in Table 2. Among all included patients, gender was prognostic for OS by the log rank test in LG-MCP group and HG-MCP group (P = 0.000, P = 0.002). Among patients stratified by PCI (< 31 vs. ≥31, P = 0.035) was prognostic for OS by the log-rank test in patients with HGMC-S. Among patients stratified by CCR (2 vs. 3, P = 0.010) was prognostic for OS by the log-rank test in patients with LG-MCP. Among patients stratified by HIPEC (Yes vs. No, P = 0.019) was prognostic for OS by the log-rank test in patients with LG-MCP. Overall patients stratified by pathological grade (LG-MCP vs. HG-MCP vs. HGMC-S, P = 0.000) was prognostic for OS by the log-rank test. Other factors affecting the overall prognosis included gender, CCR and HIPEC (P = 0.000, P = 0.005, P = 0.017), respectively. Similarly, CRS without HIPEC and high-grade disease showed lower PFS in univariate analysis (Fig. 1).

**Table 2:** Univariate analysis affecting overall survival after CRS

Variables	Log-rank P value of univariate analysis			
	Overall	LG-MCP	HG-MCP	HGMC-S
	P value	P value	P value	P value
Gender (male vs. female)	0.000*	0.000*	0.002*	0.804
Age (< 59 vs. ≥59, years)	0.480	0.842	0.434	0.305
Operation time (< 490 vs. ≥490, min)	0.061	0.333	0.153	0.965
Blood loss (< 1500 vs. ≥1500, ml)	0.329	0.056	0.424	0.991
PCI (< 31 vs. ≥31)	0.385	0.729	0.144	0.035*
CCR (2 vs. 3)	0.005*	0.010*	0.096	0.111
HIPEC (yes vs. no)	0.032*	0.019*	0.533	0.262
HIPEC drug regimen (MMC vs. DDP vs. MMC + DDP)	0.205	0.065	0.847	0.381
PSC (yes vs. no)	0.512	0.893	0.412	0.483
Previous surgery (yes vs. no)	0.493	0.794	0.529	0.312
Pathological grade (LG-MCP vs. HG-MCP vs. HGMC-S)	0.000*			

*CRS* cytoreductive surgery, *PCI* peritoneal cancer index, *CCR* completeness of cytoreduction, *HIPEC* hyperthermic intraperitoneal chemotherapy, *MMC* mitomycin, *DDP* cisplantin, *PSC* previous systemic chemotherapy, *LG-MCP* low-grade mucinous carcinoma peritonei, *HG-MCP* high-grade mucinous carcinoma peritonei, *HGMC-S* high-grade mucinous carcinoma peritonei with signet ring cells

*P < 0.05

### Multivariate analysis

Multivariate analysis showed that CRS without HIPEC and high pathological grade were independent risk factors for poor prognosis (P = 0.007, P = 0.000), respectively (Table 3). These two factors were remained significantly associated with PFS (P = 0.000, P = 0.000) (Table 4).

**Table 3:** Multivariate analysis affecting overall survival

Variables	B	SE	Wald	P value	Exp(B)	95.0% CI for Exp(B)	
						Bottom	Upper
Gender	− 0.173	0.153	1.287	0.257	0.841	0.623	1.134
CCR	0.223	0.129	2.998	0.083	1.250	0.971	1.608
HIPEC	− 0.191	0.071	7.156	0.007	0.826	0.718	0.950
Pathological grade	0.763	0.082	86.492	0.000	2.144	1.826	2.518

*CCR* completeness of cytoreduction, *HIPEC* hyperthermic intraperitoneal chemotherapy, *B* unstandardized coefficient, *SE* standard error, *Exp* odds ratio, *CI* confidence intervals

**Table 4:** Univariate and multivariate analysis affecting progression-free survival

Variables	Univariate log-rank *P*				Multivariable Cox regression *P*
	Overall	LG-MCP	HG-MCP	HGMC-S	
Treatment	0.000*	0.000*	0.153	0.187	0.000*
CRS					
CRS + HIPEC					
Pathological grade	0.000*				0.000*
LG-MCP					
HG-MCP					
HGMC-S					

*CRS* cytoreductive surgery, *HIPEC* hyperthermic intraperitoneal chemotherapy, *LG-MCP* low-grade mucinous carcinoma peritonei, *HG-MCP* high-grade mucinous carcinoma peritonei, *HGMC-S* high-grade mucinous carcinoma peritonei with signet ring cells

*P < 0.05

### Complication analysis

Among all patients, 73(14%) had intestinal fistula, 15(3%) had abdominal hemorrhage, 12(2%) had urinary fistula, and 5(1%) had incision dehiscence. The overall incidence of grade III-IV complications was 20%. Perioperative deaths occurred in 6 patients, which were all related to postoperative complications. The incidence of grade V complications was 1%. All patients had no complications related to HIPEC. The largest international sample size study on CRS and HIPEC in the treatment of PMP reported that the incidence of major complications was 24%, and the perioperative mortality rate was 2% [11].

## Discussion

The patients included in this study were confirmed as appendiceal pseudomyxoma peritonei by two experienced pathologists. In 2016, Peritoneal Surface Oncology Group International (PSOGI) divided PMP into four categories [12], as acellular mucin (AC), LG-MCP, HG-MCP, and HGMC-S. Their definition on PMP were regarded as a milestone [13]. All patients with AC had reached CCR 0–1, so our study analyzed the other three pathological types.

This paper mainly analyzed the effect of HIPEC in patients with appendiceal pseudomyxoma peritonei, and systematically clarified the factors affecting the prognosis and progression-free of PMP. PMP is a clinical syndrome characterized by the progressive accumulation of mucinous ascites within the peritoneal cavity [14]. The primary tumor is most commonly a perforated appendiceal mucinous tumor, but can also occur from other tumors such as ovarian, gastric, or colorectal cancers [15, 16]. The disease usually takes an indolent course with limited cases of lymph node or liver metastases reported [17–19]. According to the inclusion criteria, 526 patients were included in our study.

The median age of patients at hospitalization was 59 years (range 27–85), and the incidence rate of female was higher than that of male (57% vs. 43%). Most patients were diagnosed in local hospitals without receiving normative surgery. In these cases, the patients attended to our center for treatment only if they had enormous tumor burden. To some extent, this limits complete cytoreduction rather than operative skill. Other reports revealed that the small intestine was widely involved, the length of small intestine that be retained did not meet physiological and nutritional needs after operation, or the hilar structure was invaded, and the tumor cannot be completely removed [20–23]. PCI is a recognized index to evaluate tumor load worldwide. Extensive PMP is defined as the PCI ≥ 28 [21]. The median score of PCI in this paper was 31 points. Univariate analysis showed that PCI ≥ 31 can be used as a risk factor for poor prognosis in HGMC-S, and CCR 3 showed poor postoperative survival in LG-MCP compared with CCR 2. The results of a multicenter study on Peritoneal Surface Oncology Group International indicated that high peritoneal cancer index (P = 0.013) and debulking surgery (CCR, 2 or 3; P < 0.001) as independent predictors for a poorer progression-free survival [11]. Although PCI and CCR showed no survival difference in HG-MCP patients. Nevertheless, we recommend that a clear follow-up plan be developed after CRS and HIPEC to detect recurrence and plan following treatment.

In a present study, Narasimhan et al. reported a single-institution 10-year experience in management of appendiceal PMP with CRS and HIPEC. In their entire cohort, HIPEC was used in cases that had an incomplete cytoreduction. High-grade histology was a prognostic factor for a worse overall survival [23]. A previous study form Helsinki University Center Hospital reported 56(64%) patients received HIPEC (median PCI 20, range 5–29), 12(14%) were treated non-radically in an attempt at HIPEC (median PCI 34.5, range 29–39) [24]. However, the above two authors did not analyze the effect of HIPEC on prognosis. Glehen et al. analyzed the prognosis for 174 cases of PMP with non-radical resection. The results showed that HIPEC and repeated surgical resection were the factors to improve the survival of patients, and HGMC-S and lymph node metastasis were the significant risk factors affecting the prognosis [25]. A multicenter study evaluated the prognostic effect of HIPEC with CRS, compared with CRS alone, in patients with PMP. Their result demonstrated that the CRS-HIPEC group had better 5-year overall survival in CC-2/3 (16.1% [95% CI, 10.4–24.8%] vs. 28.4% [95% CI, 19.6–41.1%]; P = 0.007) [26]. High-grade histology was found to be independently associated with worse overall survival and progression-free survival. This is not unexpected, as other series also reporting histological grade influencing survival and progression-free survival [21, 22]. Other statistics revealed that male presented a worse prognosis in patients with LG-MCP and HG-MCP. These conclusions were consistent with the other two reports [27, 28].

In this study, we mainly focused on the role of HIPEC in different pathological types. In comparison of CRS and CRS + HIPEC in different pathology group, LG-MCP group had significant statistical differences in both postoperative survival and progression-free survival. It was indicated that HIPEC could prolong postoperative survival and progression-free survival of LG-MCP patients. Chua et al. also demonstrated that HIPEC was associated with an improved rate of progression-free survival [11]. However, there was no statistical differences in HG-MCP and HGMC-S group, which may be related to high degree of malignancy and poor prognosis. Our application of HIPEC in the treatment of HG-MCP and HGMC-S is mainly based on the control of malignant ascites, which is consistent with the reports of other scholars [21, 29]. In the present study, however, HIPEC with different drug regimens, PSC and previous surgery did not seem to be associated as much with the prognosis and progression-free survival. The same result of drug regimen in HIPEC has been elucidated in another study [11]. Likewise, the PMP response percentages to systemic chemotherapy were low [30]. In addition, previous surgery has also been proved no relevant to the prognosis and progression-free survival in PMP [6].

## Conclusions

To the best of our knowledge, this is the largest monocentric study on the role of HIPEC in patients of Chinese ethnicity with incomplete cytoreduction for appendiceal PMP. This study showed that HIPEC may prolong the survival of patients with low-grade appendiceal PMP who cannot achieve complete cytoreduction. High pathological grade indicates poor survival and rapid tumor progression. However, the role of HIPEC needs to be further verified in prospective study. In addition, patients with one residual tumor nodule and those with multiple nodules also need to be further compared in the future.

## Data Availability

The datasets generated and/or analyzed during the current study are not publicly available due to privacy or ethical restrictions but are available from the corresponding author on reasonable request.

## Abbreviations

PCI: peritoneal cancer index
CCR: completeness of cytoreduction
HIPEC: hyperthermic intraperitoneal chemotherapy
MMC: mitomycin
DDP: cisplantin
PSC: previous systemic chemotherapy
LG-MCP: low-grade mucinous carcinoma peritonei
HG-MCP: high-grade mucinous carcinoma peritonei
HGMC-S: high-grade mucinous carcinoma peritonei with signet ring cells
CRS: cytoreductive surgery
